# Self-assessed levels of preparedness, engagement willingness and teaching preferences on antibiotic use of medical and pharmacy students in Romanian universities: a cross-sectional study

**DOI:** 10.1186/s12909-024-06182-8

**Published:** 2024-10-25

**Authors:** Ioana Ghiga, Emma Pitchforth, Gabriel Adrian Popescu, Ibolya Fulop, Cecilia Stålsby Lundborg, Anna Machowska

**Affiliations:** 1 Department of Global Public Health, Karolinska Institutet, Stockholm, 171 77 Sweden; 2 Primary Care Research Group, University of Exeter Medical School, St Luke’s Campus, Heavitree Road, Exeter, EX1 2LU UK; 3 Faculty of Medicine, University of Medicine and Pharmacy “Carol Davila” Bucharest, Bucharest, Romania; 4 Faculty of Pharmacy, George Emil Palade University of Medicine, Pharmacy, Science and Technology of Targu Mures, Targu Mures, Romania

**Keywords:** Health professionals, Medical students, Pharmacy students, Education, Antibiotic prescribing, Preparedness

## Abstract

**Background:**

To effectively support health professionals in optimizing antibiotic prescribing and dispensing, policymakers need to understand how these professionals are trained, feel prepared and want to be educated. The study aimed to assess the current situation and explore potential improvements in antibiotic use among future health professionals in Romania by: (i) evaluating their self-assessed preparedness on antibiotic-related topics, (ii) understanding their perceptions of their role in antibiotic stewardship, and (iii) gathering their recommendations for optimizing antibiotic use.

**Methods:**

A survey of students’ self-assessment of technical preparedness, engagement willingness, expectations, teaching preferences, training received and evolution of situation in Romania. Overall, 41 and 38 questions were asked to medical and pharmacy students respectively. Scores were calculated for preparedness, engagement willingness and teaching preferences to enable various comparisons. Exploratory factor analysis was used to explore the questionnaire construct.

**Results:**

A total of 479 participants completed the survey- 233 medical students from 7 universities and 246 pharmacy students from 4 universities. Median overall preparedness score indicated that most students felt prepared in at least 14 questions (out of 22 for medical students, and 19 for pharmacy students). Engagement scores for medical and pharmacy students were similar (2 and 3 out of 4). Overall, more than half reported that ‘yes, very likely’ they received adequate training to ensure the appropriate use of antibiotics in their professional areas (*n* = 254, 53.5%). Medical and pharmacy students with low preparedness scores expressed a need for more education. Most of both medical and pharmacy students considered the antibiotic situation in Romania ‘will get worse’ (*n* = 159, 33.5%).

**Conclusions:**

The study’s findings have important implications for the education and training of future Romanian health professionals and highlight the need for further research on optimal and standardized tools to allow for periodic monitoring and evaluation of progress into preparedness, engagement willingness and teaching preferences on antibiotic use.

**Supplementary Information:**

The online version contains supplementary material available at 10.1186/s12909-024-06182-8.

## Background

Antimicrobial resistance (AMR) is a complex threat to global population’s health and wellbeing. Inappropriate use of antibiotics is a primary factor contributing to AMR and this is driven partially by over-prescribing by doctors or over-dispensing by pharmacists [1, 2]. To effectively support health professionals in performing their duties appropriately, policymakers would need to rely on an up-to-date and comprehensive understanding of how these professionals are trained, feel prepared and want to be educated about antibiotic use. Previous studies exploring the knowledge, attitudes, and practices (KAP) of medical and pharmacy students have often identified deficiencies in these areas [3–12]. These studies are often limited in scope by tending to focus on certain type of KAPs, that often do not account for the complexities that future healthcare workers will face in their profession – such as patient interaction, teamwork, need to stay updated on latest research in the field. A 2015 study went beyond the traditional KAP approach and assessed the preparedness of final-year medical students in Europe to prescribe antibiotics according to commonly accepted principles of prudent use and found that participating students felt the need for more education [13]. While this shed interesting findings, since 2015, there have been continuing rising trends in human antibiotic use and antimicrobial resistance in several European countries and the COVID-19 pandemic has brought a set of disruptions and challenges to the university learning system as well as increased consumption of antibiotics and may have accelerated the selection and transmission of AMR [14, 15]. Furthermore, similar self-assessments have not been undertaken among pharmacy students. Against this backdrop targeted research was undertaken among medical and pharmacy students in a country that is experiencing some of the highest burden of antibiotic use in Europe: Romania [16], with a view to obtain an up-to-date and enlarged perspective on how future Romanian health professionals feel about antibiotic use.

The study aimed to advance the understanding of the current situation and potential improvements that could be made to optimize antibiotic usage by future health professionals in Romania by: (i) learning more about how they assess their preparedness on different topics related to antibiotic use, (ii) understanding perceptions on their role and potential engagement in a range of possible activities and (iii) capturing their recommendations around antibiotic use. This further enabled the identification of certain subject-matter areas where students may want to be supported in and whether these would be particular for each participating university. Additionally, the study aimed to validate the questionnaire for future data collection efforts in a standardized manner, enabling monitoring and comparison of data collected over time.

## Methods

### Study setting and participants

The study took place in Romania where the higher education system is aligned with the Bologna process, with the medical program duration of study being six years and the pharmacy program lasting five years [17, 18]. Universities are autonomous and decide the curricula, in accordance with the national strategies and the national academic standards.

In Romania, antibiotics are prescribed only by doctors. When pharmacists consider it is appropriate – they judge there is an emergency situation and the patients cannot see a doctor, they can dispense antibiotics without prescription– usually a small amount that would allow the start of treatment.

In this cross-sectional study data was collected via questionnaire either using the electronic platform for data collection -REDCap [19] or in paper format which then was transferred to REDCap. The data collection period was between 30th October 2022 and 1st December 2022. All medical and pharmacy degree students in their final year of studies at Romanian universities were eligible to participate. The web-based link was disseminated through social media, as well as through specialised networks such as the students’ associations. The first author visited eight faculties in Romania that were in relative closed proximity to each other to perform data collection. In Romania there are currently 13 relevant universities, out of these 12 have both faculties of medicine and pharmacy – one has only a faculty of medicine. An ideal sample was calculated to be 353^1^, considering a Confidence Interval of 95%, a margin of error of 5% and the estimated population size of approximately 4250 students. Most of the responses were gathered on paper.

### Survey instrument development and testing

The survey questionnaire was informed by the preparedness questions from the earlier 2015 study among medical students, which was complimented with questions reflective of the Romanian medical and pharmacy faculties antibiotics relevant curriculum [13]. The questionnaire was developed by the first author, a native Romanian speaker with knowledge of both the Romanian academic setting and AMR. All co-authors reviewed the questionnaire, two of whom are teaching in Romanian Faculties (medical and pharmacy). Once the questionnaire was finalised in English it was translated in Romanian and tested with two graduates (medical and pharmacy) who provided recommendations on making the language more comprehensible. Changes were made accordingly, and the questionnaire was then back-translated into English and rechecked by the study team. As no changes were further flagged, the questionnaire was subsequently deployed.

The questionnaire is structured into the following domains: (a) demographics, (b) technical preparedness (22 questions for medical students and 19 questions for pharmacy students), (c) engagement willingness (4 questions), (d) expectations (2 questions), (e) teaching preferences (11 questions) and (f) two overall assessment questions on training received and evolution of situation in Romania (Supplemental Material Table S1, S4 and Figure S8).

### Data analysis

Descriptive statistics was used to describe participants’ characteristics and reported answers. University names were encoded to mitigate any discussion of superiority and prevent partiality in evaluations by those engaged in manuscript write-up.

Preparedness, engagement willingness and preference of teaching methods scores were calculated facilitating further analysis and understanding of data (Supplementary Materials Table S3).

Frequency and percentage of students with high, medium and low preparedness score were calculated. Percentages of students that need more education by preparedness score categories were presented.

Spearman (Pearson) correlation coefficients were estimated to evaluate association between preparedness score, willingness score and teaching preference score (percentage) at student level. Pearson correlation coefficients were calculated when both variables were normally distributed, while Spearman correlation coefficients were employed when at least one of the variables was ordinal. Specifically, Pearson correlations were estimated between (i) preparedness percentage and willingness percentage, (ii) preparedness percentage and teaching preference percentage, (iii) willingness percentage and teaching preference percentage. Spearman correlations were estimated between (i) preparedness score and willingness score, (ii) preparedness score and teaching preference score and (iii) willingness score and teaching preference score.

Exploratory factor analysis (EFA) was used to identify underlying factors or latent variables that explain the variation in the responses to the questionnaire [20]. Correlation coefficients between questions were calculated to assess the relationship between the questions in EFA. Bartlett’s test of sphericity [21]and the Kaiser-Meyer-Olkin (KMO) [22] were used to assess the adequacy of the data for conducting a factor analysis. The number of factors was determined based on eigenvalues greater than 1 [23]. Factor loadings for each question were calculated to determine which factors they belong to. Lastly, Cronbach’s alpha test was used to assess the internal consistency of the factors [24].

R version 4.2.1 software was used for all statistical analysis. Specifically, the R packages ‘psych’ and ‘GPArotation’ were used to perform Principal Factor Analysis on the data set. This method allows for correlation between the factors. To enhance the interpretability of the factors, the analysis was rerun with an oblique rotation (specifically ‘oblimin’). This type of rotation allows the factors to be correlated and improve the internal consistency reliability of the factors.

Content analysis using an Excel table, was used to analyse the free text expressed in the two questions that aimed to capture expectations and recommendations.

## Results

**Fig. 1:**
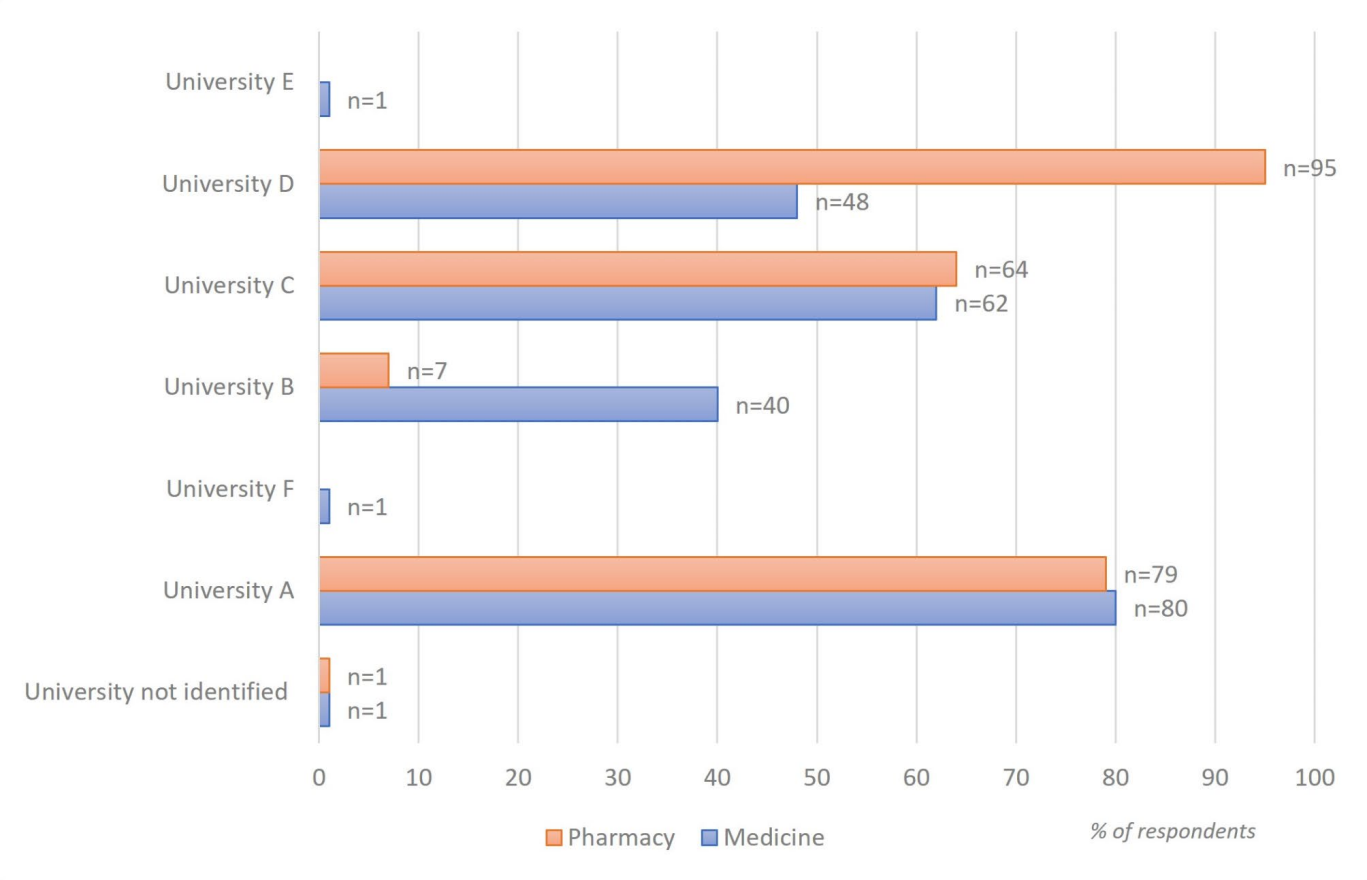
Distribution of respondents based on university and faculty

A total of 479 answers were received, 233 from medical students (from 7 universities) and 246 from pharmacy students (from 4 universities) (Fig. 1). Most of respondents studied at University A (*n* = 159, 33%), followed by respondents from University D (*n* = 143, 30%). Two respondents – one from a faculty of pharmacy and one from medical, did not specify their universities. Most of participants (95.4%) were of Romanian nationality (*n* = 457) whereas the remaining 5% had other nationalities, mainly from EU countries. Overall, most participants anticipated they will graduate this year 99.8% (*n* = 478).

A median age of responders was 24 at medical and 23 at pharmacy faculty (Supplemental Material Table S4).

### Preparedness findings

The Supplementary Material contains figures S1-5 which show responses across the five technical preparedness sub-domains across faculties.

**Fig. 2:**
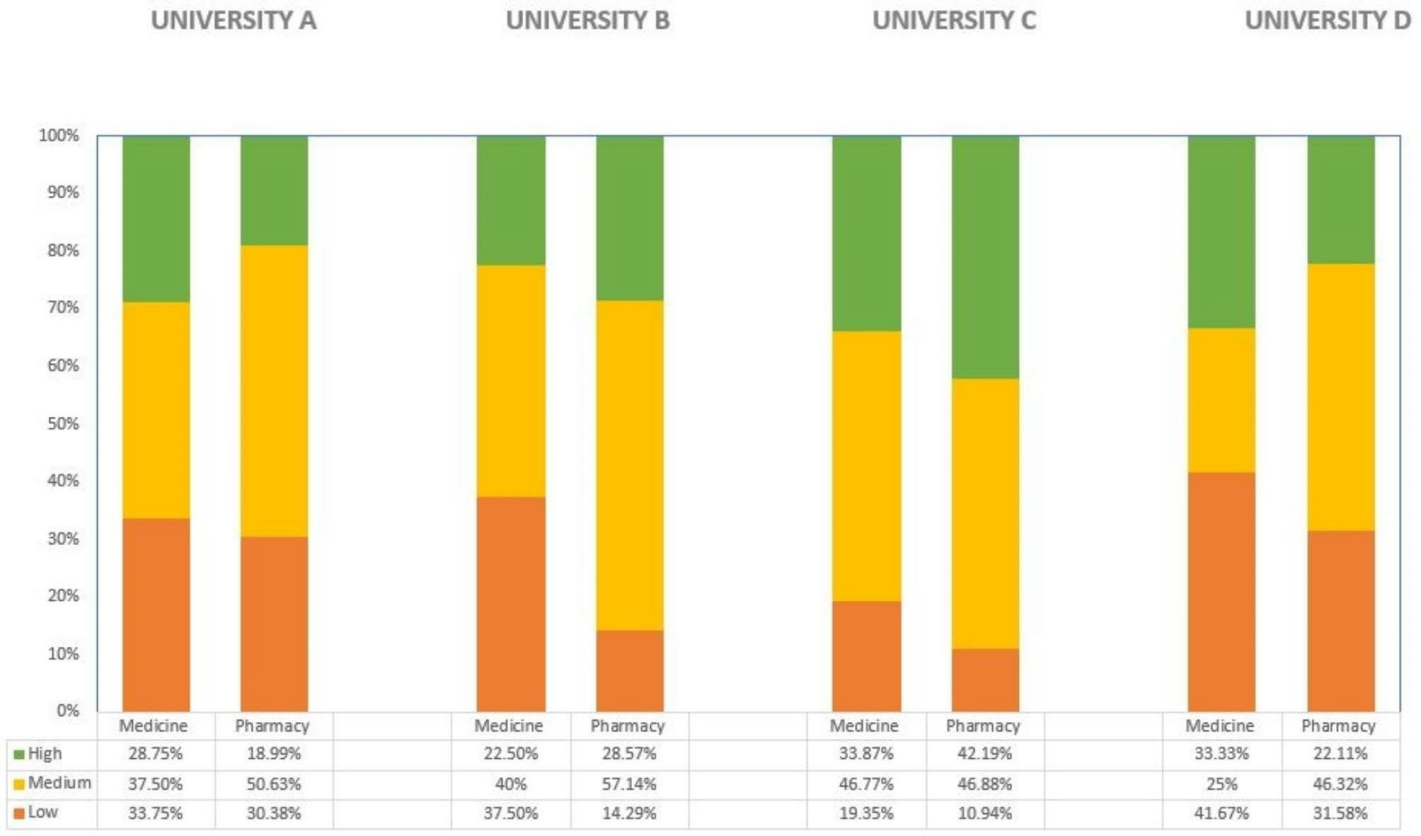
Self-assessed levels of preparedness across different universities and faculties (the two universities that had only one respondent were excluded from the analysis)

The median overall preparedness score for both medical and pharmacy students were 14, signalling that most students felt prepared in at least 14 questions. Figure 2 shows a self-assessed levels of preparedness across different universities and faculties. The two universities that had only one respondent are not reflected. The highest perceived level of preparedness is among students from the University C, with the pharmacy students feeling most prepared (42%), followed by the medical students at the same university (34%).

### Willingness findings

**Fig. 3:**
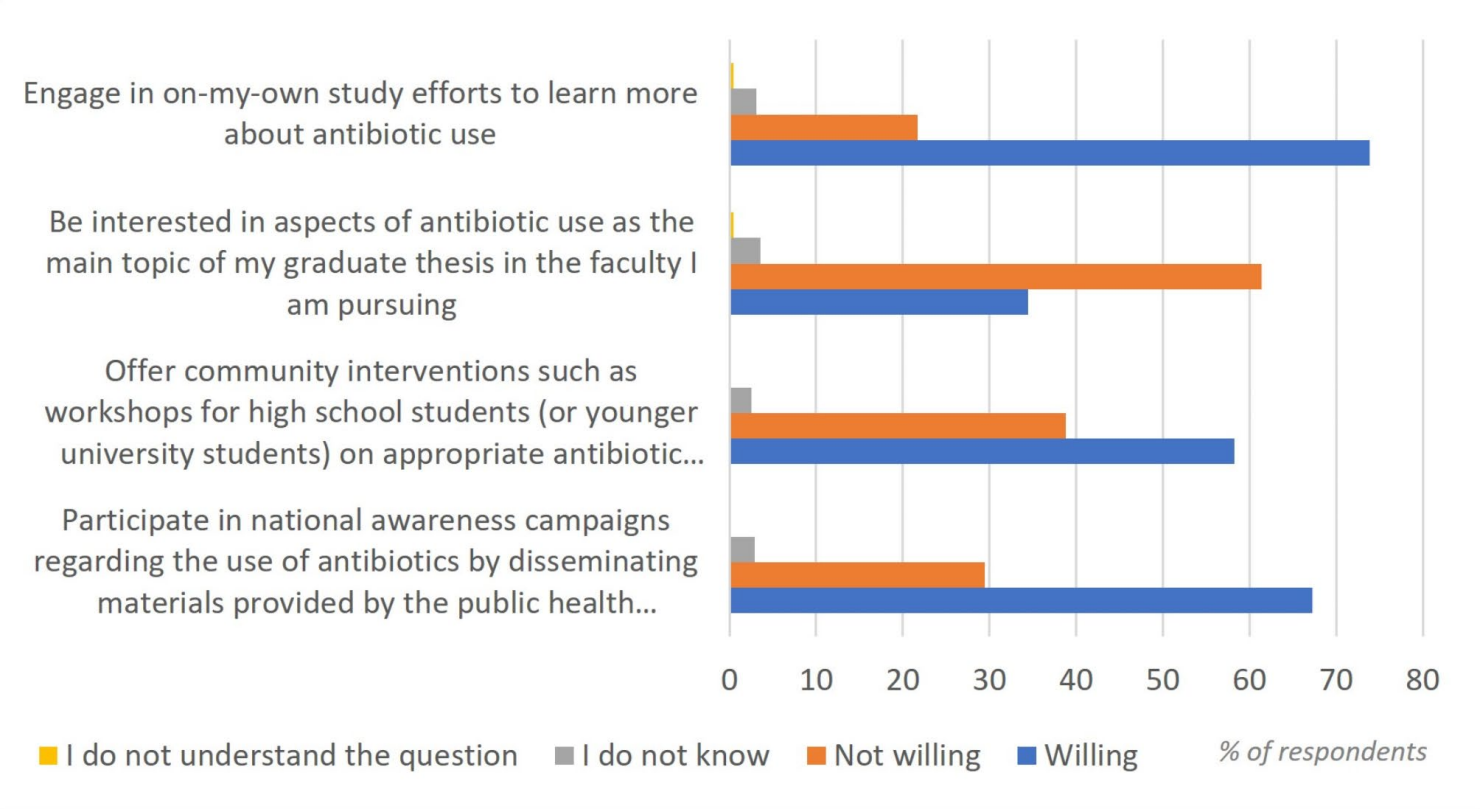
Overview of willingness to engage in different activities

The overall willingness to engage in different activities in presented in Fig. 3.

The highest willingness among participants was to engage in on-their-own study efforts and they were least willing to have their thesis graduating paper on the subject of antibiotic use. Engagement scores for medical and pharmacy students were 2 and 3.

### Preference for teaching methods

**Fig. 4:**
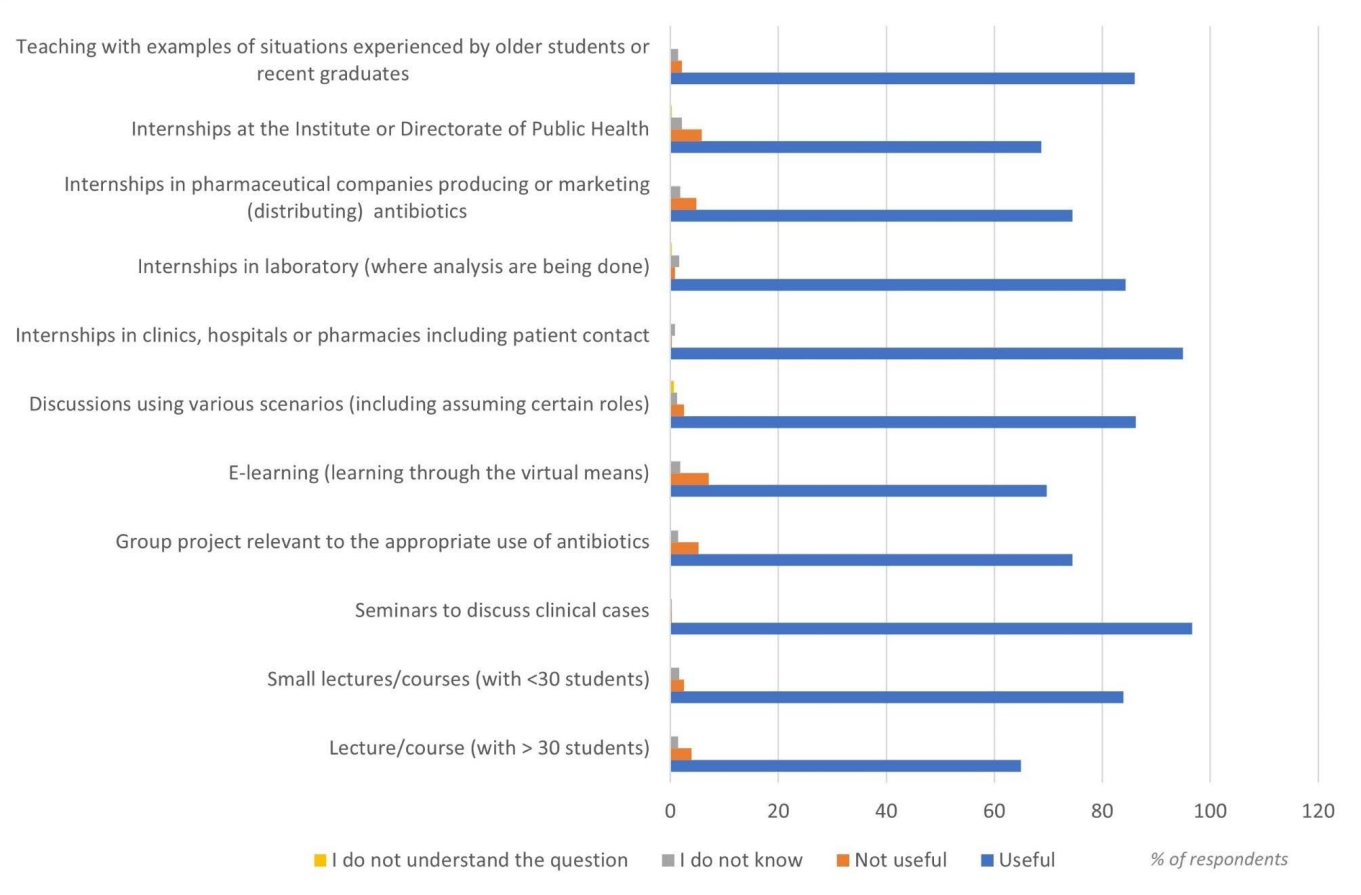
Overview of preference for teaching methods

The corresponding score found a median value of nine for medical students and 10 for pharmacy students, indicating an overall preference for most of the listed 11 teaching methods. The top three most valued methods were: seminars discussing clinical cases (96.7%), internships in clinics, hospitals, pharmacists that included contact with patients (95%) and teaching with examples of situations experienced by older students or recent graduates (86%). Figure 4 provides an overview of the overall preference for teaching methods.

No strong correlation was detected between preparedness and engagement scores, preparedness and teaching scores, engagement and teaching preference scores, percentages of preparedness and engagement, percentages of preparedness and teaching preferences, and percentages of engagement and teaching preferences.

### Participants’ expectations

169 medical students and 128 pharmacy students provided recommendations for the universities on ways to further the knowledge on antibiotics. Whereas 156 medical students and 108 pharmacy students provided recommendations to the Ministry of Health to create policies or activities aimed at ensuring the responsible use of antibiotics in Romania. Results are presented in the Supplementary Material.

### Overall assessment questions on training received and evolution of situation in Romania

**Fig. 5:**
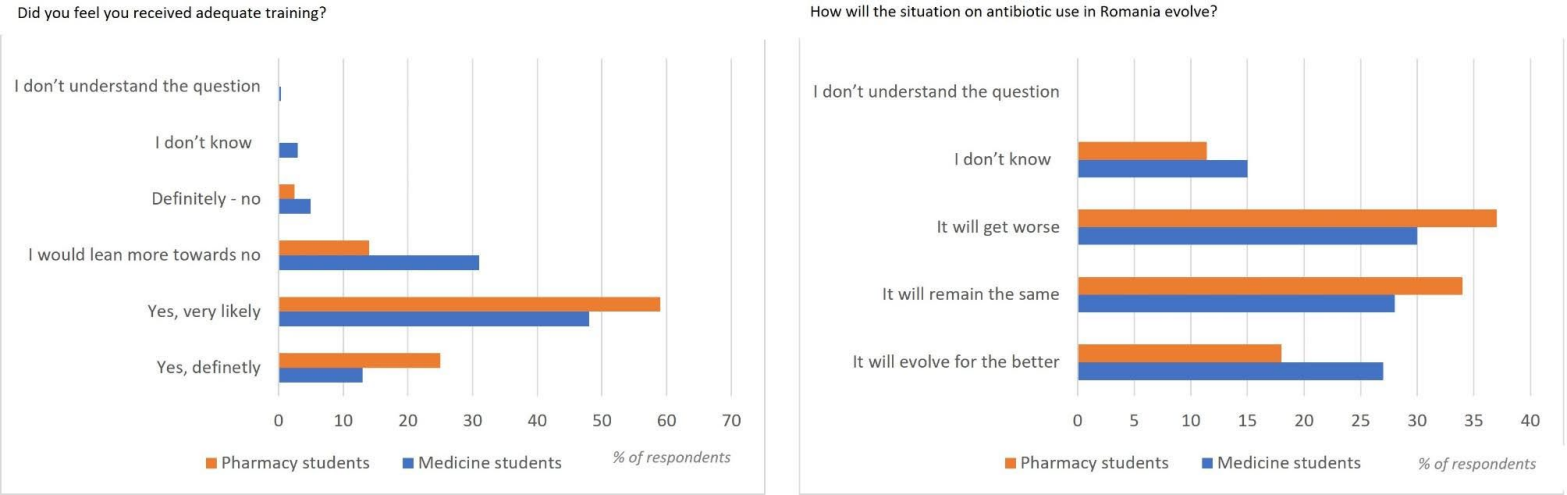
Students’ answers to the Q14 and 15

Results from the final questions are presented by student category in Fig. 5.

Overall, when asked whether they received adequate training to ensure the appropriate use of antibiotics in their professional areas and competencies: the majority reported that ‘yes, very likely’ (*n* = 254, 53.5%), followed by ‘I would lean more towards no’ (*n* = 104, 21.9%) and ‘Yes, definitely’ (*n* = 92, 19.4%).

When comparing percentages of students that need more education (answered ‘I would lean more towards no’ and ‘Definitely no’) by preparedness score categories, both medical and pharmacy students the highest proportion of students that expressed a need for more education also had low preparedness scores (medical students 20.2%, pharmacy students 8.5%).

When asked how they think the situation with the use of antibiotics in Romania will evolve, the majority considered ‘It will get worse’ (*n* = 159, 33.5%).

### Exploratory factor analysis

Correlation matrixes informed the association between the questions (Supplementary Material Figure S6). Bartlett sphericity and the KMO tests were performed to test the hypothesis of the factorability of the data; these indicated that the data are suitable for factor analysis (please see Table 1).

**Table 1:** Bartlett sphericity and the KMO tests results for medicine and pharmacy students

Test	Results medicine students	Results pharmacy students
Bartlett’s sphericity test	Measure of Sample Adequacy (MSA) = 0.767	Measure of Sample Adequacy (MSA) = 0.726
Kaiser-Meyer-Olkin (KMO)	Approx. Chi-Square = 3995.82 df = 741 Sig = 0.00	Approx. Chi-Square = 3445.327 df = 630 Sig = 0.00

Based on estimated eigenvalues seven factors for medical students’ questions and five for pharmacy students’ questions were selected (Supplementary Material Figure S7).

Factors loadings for each question were calculated and are presented in Supplemental Material Tables S5 and S9. Cumulative variances are presented in Supplemental Material Tables S6 and S10. Correlation coefficients between factors are presented in Supplemental Material Tables S7 and S11. The composition of each of the factor is shown in Supplementary Material Tables S9 (medical) and S12 (pharmacy). Cronbach’s alpha test results to assess the internal consistency of the factors are presented in Supplementary Material Table S13.

## Discussion

Our findings show that students of both medical and pharmacy, feel prepared in assessing the need for antibiotics, understanding antibiotic resistance as well as communication and engagement on the topic of antibiotic use. The findings on sufficient knowledge for assessing the need for antibiotics – such as identifying early signs of infection are consistent with previous research across several EU countries, including Romania [13], converge with findings of a systematic review of 22 studies that revealed students considered themselves prepared to identify signs of infection and making a clinical decision but at the same time, somewhat diverge with findings from the same review which found students felt less preparedness when it comes to interpretation of results [25–28]. On making decisions to initiate antibiotic treatment, the majority of medical students did not feel ready in three out of the seven areas, particularly when it came to prescribing a combination of antibiotics with other medicines, setting the appropriate timing and duration of antibiotic administration, and deciding on treatment without the use of guidelines. The recommendations students made pertaining to integration and emphasis on resistance and optimization into the curriculum, strengthen the need to enhance preparedness in these areas. These findings are also consistent with a systematic review assessment of 72 studies that revealed final year medical students have insufficient capabilities to prescribe antibiotics safely and effectively [29]. In our study, most pharmacy students felt prepared to initiate antibiotic treatment. Most studies that involve pharmacy students tend to investigate their knowledge, attitudes, and practices related to antibiotic use for viral infections and effectiveness of antibiotics, therefore it is difficult to compare our findings with other studies, although these would indicate an insufficient level of knowledge [3, 6, 7, 30–32].

Another area where students seem to feel not ready pertains to monitoring antibiotic therapy and assessing emerging evidence with both medical and pharmacy students not feeling ready to interpret the findings of scientific studies, nor participate as a researcher in clinical or epidemiological research. This is an expected finding considering the relatively small research publications output in Romania [33].

Two universities had the same patterns of preparedness among both medical and pharmacy students, whereas two others had variability. It is difficult to explain these differences, but they might be a combination of available resources and teaching methods at that particular time in different universities or cultural differences, given that Romanian universities have similar curricula.

The overall medium score level of preparedness is consistent with the majority of answers to the question on whether students felt they received adequate training to ensure the appropriate use of antibiotics in their professional areas and competencies. The majority responding ‘yes, very likely’ suggesting a carefully optimistic outlook, with areas of improvement, but an overall satisfactory base level of preparedness. However, it is worth remembering that this is a self-assessment.

No significant correlations were observed between the different scores and percentages of the question domains indicating that there was relationship between them. This is a somewhat surprising finding, as it would have been expected the higher preparedness scores, with a particular view of the communication and engagement, might be associated with higher score on engagement willingness as these contain activities that relate to engagement in community activities and national awareness campaigns. Similarly, certain teaching preferences such as desire to pursue a certain type of internship – with the public health specialized direction- might be associated with higher scores on engagement willingness. Based on our data, teaching preferences therefore are not related to engagement willingness.

This study also sheds light on the commonalities and differences in perception of preparedness levels between medical and pharmacy students. Interdisciplinary collaboration is needed to address the challenges posed by misuse of antibiotics. However, to promote such collaboration, programs need to be built on an understanding of common needs. While acknowledging the different roles of these professions, an area where more in-depth and joint training might be explored is in relation to monitoring antibiotic therapy and assessing emerging evidence in particular in engaging in research activities – whether these are clinical or public health. This is because both categories of students felt unprepared and these subjects would equip all students with relevant knowledge to engage and critically assess research which is relevant for both professions.

Education and training play a critical role in shaping professional attitudes and behaviors. The recommendations to both universities and Ministry of Health reveal potential values and norms for Romanian students such as responsibility – with both groups of students recognizing the importance of responsible us of antibiotics, collaboration – although mostly focused within their profession and with patients, critical thinking reliant on evidence-base practice and patient-centered care and continuous learning. These values and norms could shape future attitudes and behaviours of healthcare professionals. Across the different categories of students, there was a consistent theme on the importance of greater agency, interaction and engagement. Furthermore, when asked about willingness to engage in different activities, most responders chose ‘on their own efforts’. Social cognitive theory, which focuses on the individual cognitive processes that underlie learning and behavior, stresses the role of the learner in constructing their own understanding and knowledge, self-efficacy as well as the importance of observations and modeling [34, 35]. Previous research found that medical students who scored higher on measures of empathy and professionalism during their training were more likely to exhibit these qualities as practicing physicians [36]. Social cognitive theory also postulates a greater likelihood of adopting a certain behaviour, when it is believed that one can perform it successfully (self-efficacy) and when a positive outcome is expected. This theoretical framework also recognizes the behavioural impact of social and environmental factors, with a greater predisposition of individuals to engage in activities that are perceived to be socially acceptable or normative such as collaborative practice in enhancing patient care and improving health outcomes [37, 38]. Considering these theoretical underpinnings and our findings, several interventions could be implemented. With the overall aim of enhancing preparedness and confidence, these could consist of: (i) evidence-based training with an emphasis of enhancing skills to critically evaluate research studies, (ii) intra- and inter-professional education based on shared goals and joint training programmes, to bring greater understanding on different healthcare workers’ roles and responsibilities, and enhance collaboration, teamwork and communication and coordination in patient care; (iii) simulations and role-playing exercises to gain practical experience in a controlled environment, (iv) mentorship programmes to support modeling of students’ behaviours based on professionals that are aware they are serving this role.

Lastly, our findings have methodological implications. Previous research highlighted the difficulties in engaging in systematic analysis in this field due to lack of standardization of data collection instruments, whether they pertain to analysis of KAP surveys [25] or effectiveness of interventions [39].

While our instrument has proven valuable in capturing a rich information dataset relevant for a Romanian setting, the results from the EFA indicated suboptimal validity and reliability of the questionnaire’s construct. Following the originally suggested 7-factor and 5-factor constructs with their respective allocated questions would not be meaningful due to significant overlaps in concepts. Therefore, a redesign and testing of the questionnaire are needed to ensure its effectiveness in capturing accurate data on students’ perceptions and preparedness related to antibiotic usage. Within the current limited dataset, Confirmatory Factor Analysis was not feasible, highlighting the need for a larger sample size in future studies to enhance the statistical robustness and generalizability of findings.

### Strengths and limitations

This study contributes significantly to the understanding of antibiotic usage preparedness among future healthcare professionals in Romania. The findings highlight nuanced differences between medical and pharmacy students in their perceived preparedness across various domains related to antibiotic prescribing and usage. Moreover, the study identifies critical areas where educational interventions could enhance students’ capabilities, particularly in interpreting scientific evidence and engaging in interdisciplinary collaboration. The emphasis on social cognitive theory provides a theoretical framework that enhances the study’s relevance to professional behavior and educational strategies. However, several limitations should be considered when interpreting the findings of this study. Firstly, the reliance on self-reported data for assessing students’ preparedness may introduce bias, as participants may overestimate or underestimate their abilities. Secondly, the study’s cross-sectional design limits its ability to establish causal relationships between educational interventions and preparedness outcomes. Thirdly, the sample size and composition, drawn from a select number of universities in Romania, may not fully represent the diversity of educational experiences and perspectives across the country. Although the questionnaire underwent validation processes, it may still require further refinement such as shortening it based on the relevant factors and using the same scale for all questions. Finally, the study’s focus on student perceptions and readiness may not fully capture actual behaviors in clinical settings post-graduation. Future research could address these limitations by incorporating longitudinal designs, expanding the sample size, and incorporating objective measures of performance in clinical practice.

## Conclusion

Findings provide valuable information for policymakers to make informed decisions about areas where additional education and training is needed for future or recent-graduated health professionals. These findings can concretely inform the design of interventions that effectively enhance students’ knowledge and skills in antibiotic stewardship, thereby promoting responsible antimicrobial use across healthcare settings. The study also underscores the necessity of establishing a standardized tool to assess antibiotic use preparedness consistently among medical and pharmacy students. Such a tool would ensure the reproducibility of research outcomes across diverse educational and cultural contexts, facilitating comparisons and improvements in educational strategies aimed at combating antimicrobial resistance.

## Electronic supplementary material

Below is the link to the electronic supplementary material.

Supplementary Material 1

## Data Availability

Data is provided within the manuscript and supplementary information files.

## Footnotes

1 There is inconsistent reporting on graduation figures, therefore we use the admission data as a proxy for graduation. Based on 2018 data from University of Medicine and Pharmacy “Carol Davila” Bucharest – a graduation rate is estimated at 92.4% (calculated by dividing 1223 graduates in 2018 to 1324 initially accepted 6 years earlier). Therefore, an approximate total target population is 4250.

## Abbreviations

AMR: Antimicrobial Resistance
EFA: Exploratory Factor Analysis
EU: European Union
KAP: Knowledge, Attitudes, and Practices
KMO: Kaiser-Meyer-Olkin
NBCMMD: National Bioethics Committee for Medicals and Medical Devices
