# Supplementary material for: Self-assessed levels of preparedness, engagement willingness and teaching preferences on antibiotic use of medical and pharmacy students in Romanian universities: a cross-sectional study
Source: BMC Med Educ. 2024 Oct 25;24:1214. doi: 10.1186/s12909-024-06182-8 (PMC11520128; doi:10.1186/s12909-024-06182-8)
Supplement: Supplementary file 1 — Supplementary Material 1 [file 12909_2024_6182_MOESM1_ESM.docx]

**Supplementary materials**

[Results of the content analysis of students’ expectations – Question 12 a) and b) 2](#_Toc149924856)

**LIST OF TABLES**

[Table S1 Overview of questions per faculty and corresponding question labels 4](#_Toc174527532)

[Table S2 Questions labels 5](#_Toc174527533)

[Table S3 Methods for calculating scores 8](#_Toc174527534)

[Table S4 Age of participants by faculties 8](#_Toc174527535)

[Table S5 Factor loadings for the seven-factor model for medicine students’ questions 9](#_Toc174527536)

[Table S6 Summary of loadings and cumulative variances for medicine students’ questions 10](#_Toc174527537)

[Table S7 Correlation coefficients between factors for medicine students’ questions 11](#_Toc174527538)

[Table S8 Which questions belong to each factor for medicine students’ questions 11](#_Toc174527539)

[Table S9 Factor loadings for the five-factor model for pharmacy students’ questions 12](#_Toc174527540)

[Table S10 Summary of loadings and cumulative variances for pharmacy students’ questions 13](#_Toc174527541)

[Table S11 Correlation coefficients between factors for pharmacy students’ questions 13](#_Toc174527542)

[Table S12 Which questions belong to each factor for pharmacy students’ questions 14](#_Toc174527543)

[Table S13 Cronbach’s alpha test results for medicines and pharmacy students’ questions 14](#_Toc174527544)

***LIST OF FIGURES***

[Figure S1 Overview of responses across faculties for preparedness to assess the need for antibiotics 14](#_Toc174527545)

[Figure S2 Overview of responses across faculties for preparedness to make decisions on initiating antibiotic treatment 16](#_Toc174527546)

[Figure S3 Overview of responses across faculties for preparedness to monitor antibiotic therapy and assessing emerging evidence 17](#_Toc174527547)

[Figure S4 Overview of responses across faculties for preparedness on antibiotic resistance 17](#_Toc174527548)

[Figure S5 Overview of responses across faculties for preparedness for communication and engagement activities related questions 18](#_Toc174527549)

[Figure S6 Correlations between questions relevant for medicine and pharmacy students 19](#_Toc174527550)

[Figure S7 Overview of Eigenvalues of factors for medicine and pharmacy students 19](#_Toc174527551)

[Figure S8 Survey text and questionnaire 21](#_Toc174527552)

# *Results of the content analysis of students’ expectations – Question 12 a) and b)*

- Recommendations for the universities on ways to further the knowledge on antibiotics

Medical students’ views were in this respect were grouped into three major categories. These are:

1. **More frequent practical experience**: through clinical internships, workshops, and case simulations. student-led circles organized with a limited number of participants. Some students expressed a desire for greater interaction with patients and enhanced collaboration with the teaching assistants and other healthcare professionals to establish therapy, while being given more autonomy to make therapeutic decisions for patients (with the mention that this would include a checked step by a supervisor) instead of passively assisting in internships.
2. **Improved teaching methods**: by making them more interactive and practical, including the use of group projects, debates, extracurricular activities, and selected clinical cases taught in other specialties, using mnemonics to facilitate the learning process, emphasizing knowledge of common doses and indications for frequently used antibiotics, and teaching a greater number of courses that emphasize the mechanisms and the need for prescriptions. The students considered teaching should also include the commercial names, schematized courses, prescriptions for at least common antibiotics and annual updates on new medicals and any modifications to old ones. Summary sheets that are easy to understand, and provide clear, easily understandable information that emphasizes clinical relevance.
3. **Integration and emphasis on antimicrobial resistance and optimization into the curriculum**: participants recommending an emphasis on the impact of resistance, especially in Romania, the risks of antibiotic resistance, and the use of optimized guidelines; clearer schemes regarding the type of antibiotics used for each type of infection and involved bacteria and what is used as a second-line treatment. Another recommendation was an increased involvement of the pharmacology department in presenting clinical cases and including longer pharmacology courses of at least three semesters. Optional courses that address antibiotics at a higher level and dedicated seminars for antibiotics and antivirals were also highlighted. Similarly, more in-depth immunology and microbiology courses were stressed.

Pharmacy students’ views were in this respect were grouped into three major categories. These are:

1. **Enhanced curriculum design and content**: develop a comprehensive curriculum that covers the basics of antibiotics, their mechanism of action, appropriate use including in specific patient populations, such as pregnant women and children, and antibiotic resistance. Emphasize the importance of antibiotic stewardship and rational use of antibiotics, including the risks associated with antibiotic misuse and the consequences of antibiotic resistance. Use up-to-date and evidence-based resources when teaching about antibiotics. Lengthen the time dedicated to these courses – e.g. microbiology should be studied for one year, not just one semester, disciplines such as semiology and pathology should also focus on the study of infectious diseases, allocating more hours for teaching to deepen the material without rushing the learning process. Creating optional courses on antibiotics, treating animals with antibiotics, and development of antibiotic resistance.
2. **Incorporate practical and interactive learning approaches**: such as clinical cases, workshops, quizzes, case simulations, internships in clinics, to facilitate active learning and engagement.
3. **Encourage scientific participation**: provide opportunities for students to participate in research related to antibiotic use and antibiotic resistance and encourage them to attend conferences and seminars related to antibiotics and antibiotic resistance.

- Recommendations for the Ministry of Health to create policies or activities aimed at ensuring the responsible use of antibiotics in Romania

Medical students’ views were in this respect were grouped into five major categories. These are:

1. **A supportive environment for education and awareness**: many of the suggestions relate to raising awareness among the public, healthcare providers, and students about the appropriate use of antibiotics, the risks of resistance, and the difference between bacterial and viral infections. These include retraining family doctors, organizing educational campaigns in schools, universities, implementing information campaigns through media, and creating public education programs, creating virtual platforms to offer support in understanding antibiotic administration and promoting medical education. One suggestion was to also incorporate pharmacological education into high school curriculums.
2. **Regulation and enforcement**: several suggestions involve regulatory measures to control the sale and dispensing of antibiotics. These include prohibiting the sale of antibiotics without a prescription (without exceptions), enforcing stricter control of antibiotic prescription, and dispensing, and creating national protocols for antibiotic administration. Recommendation was made for better control of pharmacies to prevent patients from having many antibiotics at home. There was also a suggestion that not all doctors should be allowed to prescribe antibiotics and that only those who have completed relevant courses should be authorized to do so.
3. **Collaboration with healthcare providers**: some suggestions focus on working collaboratively with healthcare providers, such as family doctors and school doctors, to promote responsible antibiotic use. These include providing annual courses for family doctors on antibiotic use, assigning a responsible doctor for antibiotic administration, and conducting epidemiological studies related to bacterial resistance in Romania.
4. **Campaigns and advertisements**: several suggestions involve creating campaigns and advertisements to promote responsible antibiotic use, including advertising campaigns through accessible personalities, mass media campaigns, and campaigns in family doctors' offices. The mentioned media outlets were TV, billboards, and social media to ensure maximum exposure. Campaigns should also emphasize prevention recommendations such as improving hygiene practices and increasing vaccination rates to reduce the need for antibiotics.
5. **Strengthening epidemiological surveillance to monitor antibiotic resistance trends**: some suggestions focus on the need for monitoring and collecting data related to antibiotic resistance, such as conducting statistics on patterns of resistance to last-resort drugs and creating a special document to note the type and dose of antibiotics received by each patient throughout their life (individual consolidated registry). Other suggestions consisted of establishing committees to oversee the judicious use of antibiotics in hospitals as well as and monitoring of family doctors prescribing patterns. The need for more epidemiologists was also highlighted.

Pharmacy students’ views were in this respect were grouped into three major categories. These are:

1. **Increasing awareness and education about antibiotic resistance and the responsible use of antibiotics**: among various groups, such as the general population, doctors, pharmacists, and students. The most common suggestions include carrying out national awareness campaigns in schools, hospitals, workplaces, introducing this topic into health education classes, creating informative videos for TV, radio and social media. The imposition of courses on antibiotics in universities not related to the health field to ensure that people understand when antibiotics are necessary and when they are not, was suggested.
2. **Stricter controls and monitoring of antibiotic use**: including fining pharmacies and for releasing antibiotics without a prescription and doctors for prescribing antibiotics unnecessarily, carrying out checks on family doctors and pharmacies, introducing electronic prescriptions to better monitor their use.
3. **Healthcare provision optimization**: mandatory imposition of antibiograms and increasing communication between pharmacists and doctors. Other ideas included and providing free doctors’ consultations to citizens^[[1]](#footnote-2)^ to encourage responsible use and allowing pharmacists to prescribe antibiotics and remunerating them for their expertise in this area.

Table S1 Overview of questions per faculty and corresponding question labels

| **Domain** | **Subdomains** | **Faculty** | |
| --- | --- | --- | --- |
|  |  | Medicine | Pharmacy |
| **Demographic questions** | | 8 datapoints | |
| **Technical preparedness** | Assessing the need for antibiotics | 5 questions  (Qs: 8-12) | 4 questions  (Qs: 30-33) |
|  | Decision to initiate antibiotic treatment | 7 questions  (Qs: 13-19) | 5 questions  (Qs: 34-38) |
|  | Monitoring antibiotic therapy and assessing emerging evidence | 5 questions  (Qs: 20-24) | 5 questions  (Qs: 39-43) |
|  | Antibiotic resistance | 2 questions  (Qs: 25-26) | 2 questions  (Qs: 44-45) |
|  | Communication and engagement | 3 questions  (Qs: 27-29) | 3 questions  (Qs: 46-48) |
| **Engagement willingness** | | 4 questions  (Qs: 49-52) | |
| **Expectations** | | 2 questions  (Qs not coded for statistical analysis – free text answers) | |
| **Teaching methods used for antibiotic education** | | 11 questions  (Qs: 53-63) | |
| **Assessment of overall received training** | | 1 question  (Q: 64) | |
| **Assessment of antibiotic use in RO** | | 1 question  (Q: 65) | |

Table S2 Questions labels

| **Original_Question** | **Question_number** |
| --- | --- |
| ID | ID |
| Survey.Timestamp | Survey.Timestamp |
| Age | Age |
| Romanian_citizenship | Romanian_citizenship |
| Graduation_studies_next_year | Graduation_studies_next_year |
| University | University |
| Faculty | Faculty |
| Understand.a.clinical.situation.where.the.administration.of.antibiotics.may.be.necessary | Question_8 |
| Assess.the.clinical.severity.of.an.infection | Question_9 |
| Interpret.biomedical.markers.of.inflammation | Question_10 |
| Decide.on.the.need.for.an.antibiogram | Question_11 |
| Interpret.microbiological.investigations | Question_12 |
| Differentiate.between.bacterial.colonization.and.infection | Question_13 |
| Differentiate.between.bacterial.and.viral.upper.respiratory.tract.infections | Question_14 |
| Decide.on.appropriate.empiric.treatment.without.using.guidelines | Question_15 |
| Establish.the.appropriate.timing.and.duration.of.antibiotic.administration..e.g..based.on.the.urgency.of.different.scenarios.or.specific.infection.characteristics. | Question_16 |
| Prescribe.antibiotics.in.line.with.national.guidelines | Question_17 |
| Assess.potential.antibiotic.allergies.or.other.antibiotic.related.adverse.events | Question_18 |
| Prescribe.a.combination.of.antibiotics.with.other.medications | Question_19 |
| Decide.on.continuation.or.discontinuation.of.antibiotic.treatment.in.an.optimal.timeframe.based.on.emerging.evidence.and.developments..e.g..clinical.progress.and.analysis.results. | Question_20 |
| Investigate.the.causes.of.potential.failures.of.antibiotic.treatments | Question_21 |
| Decide.on.changing.the.route.of.antibiotic.administration..for.example..from.IV.to.oral. | Question_22 |
| Participate.as.a.researcher.in.clinical.or.pidemiological..public.health..research.on.antibiotics | Question_23 |
| Interpret.the.findings.of.scientific.studies.on.antibiotics | Question_24 |
| Use.the.knowledge.gained.about.the.mechanisms.of.antibiotic.resistance.formation.to.try.to.mitigate.this.phenomenon | Question_25 |
| Engage.in.discussions.with.my.peers..or.other.colleagues..about.the.specific.negative.consequences.of.inappropriate.antibiotic.use..including.using.recent.epidemiological.data.from.Romania | Question_26 |
| Discuss.in.lay.man.s.terms.appropriate.antibiotic.use.with.patients..especially.in.situations.when.these.may.not.be.necessary… | Question_27 |
| Justify.and.maintain.my.position.in.situations.where.I.may.feel.coherced..either.by.patients..senior.colleagues.or.other.colleagues..to.prescribe.antibiotics.when.I.consider.these.are.not.needed | Question_28 |
| Work.in.a.multidisciplinary.team.in.a.clinical.setting | Question_29 |
| Understand.a.clinical.situation.where.the.administration.of.antibiotics.may.be.necessary.1 | Question_30 |
| Based.on.the.physician.s.prescription.understand.why.a.certain.antibiotic.was.prescribed | Question_31 |
| Advise.the.patient.how.to.manage.the.symptoms.without.antibiotics | Question_32 |
| Offer.recommendations.on.intermediate.drug.alternatives..which.do.not.involve.antibiotics..and.advise.the.patient.if.it.is.necessary.to.see.a.doctor.immediately | Question_33 |
| Assess.the.seriousness.of.a.clinical.situation.and.establish.whether.emergency.antibiotic.should.be.given.to.the.patient | Question_34 |
| Make.recommendations.about.over.the.counter..OTC..medications.that.are.appropriate.for.optimizing.antibiotic.use | Question_35 |
| Evaluate.potential.antibiotic.allergies.or.other.antibiotic.related.adverse.events | Question_36 |
| Understand.the.effects.of.drug.interactions.and.potential.adverse.effects.of.some.of.these.drug.combinations | Question_37 |
| Offer.guidance.to.patients.on.infection.prevention.and.spread..for.example..advice.about.the.benefits.of.vaccination..hygiene.and.hand.washing. | Question_38 |
| Recommend.that.the.patient.continue.or.stop.antibiotic.treatment.and.refer.him.to.a.doctor.depending.on.the.patient.s.clinical.presentation | Question_39 |
| Understand.the.causes.of.potential.antibiotic.treatment.failures | Question_40 |
| Provide.expert.advice.to.healthcare.professionals.on.antibiotic.choice..dose..duration.and.dose.adjustment | Question_41 |
| Participate.as.a.researcher.in.clinical.or.epidemiological..public.health..research.on.antibiotics | Question_42 |
| Interpret.the.findings.of.scientific.studies.about.antibiotics | Question_43 |
| Use.the.knowledge.gained.about.the.mechanisms.of.antibiotic.resistance.formation.to.try.to.mitigate.this.phenomenon.1 | Question_44 |
| Engage.in.discussions.with.my.peers..or.other.colleagues..about.the.specific.negative.consequences.of.inappropriate.antibiotic.use..including.using.recent.epidemiological.data.from.Romania.1 | Question_45 |
| Discuss.in.lay.man.s.terms.with.patients.appropriate.antibiotic.use..especially.in.situations.when.these.may.not.be.necessary… | Question_46 |
| Justify.and.maintain.my.position.in.situations.where.I.may.feel.coherced..either.by.patients..senior.colleagues.or.other.colleagues..to.release.antibiotics.without.prescriptions.when.I.consider.them.unnecessary | Question_47 |
| Work.in.a.multidisciplinary.team.in.a.clinical.setting.1 | Question_48 |
| Participate.in.national.awareness.campaigns.regarding.the.use.of.antibiotics.by.disseminating.materials.provided.by.the.public.health.departments | Question_49 |
| Offer.community.interventions.such.as.workshops.for.high.school.students..or.younger.university.students..on.appropriate.antibiotic.use.in.my.spare.time | Question_50 |
| Be.interested.in.aspects.of.antibiotic.use.as.the.main.topic.of.my.graduate.thesis.in.the.faculty.I.am.pursuing | Question_51 |
| Engage.in.on.my.own.study.efforts.to.learn.more.about.antibiotic.use | Question_52 |
| Lecture.course..with…30.students. | Question_53 |
| Small.lectures.courses..with..30.students. | Question_54 |
| Seminars.to.discuss.clinical.cases | Question_55 |
| Group.project.relevant.to.the.appropriate.use.of.antibiotics | Question_56 |
| E.learning..learning.through.the.virtual.means. | Question_57 |
| Discussions.using.various.scenarios..including.assuming.certain.roles. | Question_58 |
| Internships.in.clinics..hospitals.or.pharmacies.including.patient.contact | Question_59 |
| Internships.in.laboratory..where.analysis.are.being.done. | Question_60 |
| Internships.in.pharmaceutical.companies.producing.or.marketing..distributing…antibiotics | Question_61 |
| Internships.at.the.Institute.or.Directorate.of.Public.Health | Question_62 |
| Teaching.with.examples.of.situations.experienced.by.older.students.or.recent.graduates | Question_63 |
| Adequate_training. | Question_64 |
| How.do.you.think.the.situation.with.the.use.of.antibiotics.in.Romania.will.evolve. | Question_65 |
| Complete | Question_66 |

Table S3 Methods for calculating scores

| **Type of score** | **Method for calculating score** | **Further categorization** |
| --- | --- | --- |
| **Preparedness score** | Preparedness score was estimated by dichotomizing the response scale of each question as:   - - 1 – (I feel ready, I feel very ready)   - 0 – (I don't feel ready at all, I don't feel quite ready)   Values were added up for each respondent calculating individual scores. | The following thresholds were used to structure score:   - High (>= 17) - Medium (12-16) - Low (<=11)   These were chosen as for medicine and pharmacy students, the maximum possible score is 22 and 19, respectively and the cut-off thresholds should be consistent among both groups of students to aid analysis. |
| **Engagement willingness score** | Engagement willingness score was estimated by dichotomizing the response scale of each question as:   - 1 – (I am willing, I am very willing) - 0 – (I'm not willing at all, I'm not really willing)   Values were added up for each respondent calculating individual scores. | N/A |
| **Teaching methods preferred for antibiotic education** | Teaching methods score will be estimated by dichotomizing the response scale of each question as:   - 1 – (I find it useful; I find it very useful) - 0 – (I don't find it useful at all, I don't think it's very useful)   Values were added up for each respondent calculating individual scores. | N/A |

Table S4 Age of participants by faculties

| **Faculty** | **missing** | **mean** | **sd** | **median** | **iqr** | **min** | **max** |
| --- | --- | --- | --- | --- | --- | --- | --- |
| Medicine | 0 | 24.50 | 1.76 | 24 | 1 | 22 | 35 |
| Pharmacy | 1 | 23.31 | 2.29 | 23 | 0 | 22 | 42 |

Table S5 Factor loadings for the seven-factor model for medicine students’ questions

|  | **PA1** | **PA2** | **PA3** | **PA4** | **PA5** | **PA6** | **PA7** |
| --- | --- | --- | --- | --- | --- | --- | --- |
| Question_8 | -0.23 | -0.32 | 0.06 | 0.24 | 0.59 | 0.14 | 0.33 |
| Question_9 | 0.40 | 0.08 | -0.12 | -0.08 | 0.50 | 0.14 | 0.01 |
| Question_10 | 0.13 | -0.19 | -0.01 | 0.32 | 0.41 | 0.40 | -0.10 |
| Question_11 | 0.17 | -0.06 | 0.06 | -0.27 | 0.53 | -0.04 | 0.27 |
| Question_12 | 0.05 | -0.07 | 0.38 | -0.06 | 0.51 | 0.34 | -0.05 |
| Question_13 | 0.13 | 0.36 | 0.10 | 0.02 | 0.47 | -0.22 | -0.07 |
| Question_14 | 0.14 | 0.21 | -0.15 | 0.06 | 0.62 | -0.01 | 0.07 |
| Question_15 | 0.38 | -0.23 | 0.17 | 0.05 | 0.07 | -0.26 | 0.52 |
| Question_16 | 0.82 | -0.08 | 0.01 | 0.05 | 0.10 | -0.14 | -0.12 |
| Question_17 | 0.39 | -0.20 | 0.01 | 0.22 | 0.09 | -0.03 | 0.27 |
| Question_18 | 0.78 | -0.08 | -0.14 | 0.02 | -0.01 | -0.02 | 0.14 |
| Question_19 | 0.77 | 0.07 | -0.03 | -0.12 | -0.07 | 0.13 | -0.05 |
| Question_20 | 0.67 | 0.16 | 0.04 | 0.15 | 0.17 | 0.18 | 0.00 |
| Question_21 | 0.54 | -0.05 | 0.31 | 0.06 | -0.13 | 0.09 | 0.06 |
| Question_22 | 0.43 | 0.44 | 0.26 | 0.05 | 0.04 | 0.15 | 0.23 |
| Question_23 | 0.06 | -0.01 | 0.57 | 0.26 | -0.21 | 0.21 | 0.09 |
| Question_24 | 0.39 | 0.14 | 0.34 | 0.26 | -0.06 | 0.24 | 0.09 |
| Question_25 | 0.01 | 0.15 | 0.05 | 0.03 | 0.09 | 0.13 | 0.81 |
| Question_26 | 0.27 | -0.05 | 0.30 | 0.34 | 0.09 | 0.21 | -0.07 |
| Question_27 | 0.19 | 0.04 | -0.11 | 0.21 | 0.20 | 0.35 | 0.16 |
| Question_28 | 0.12 | 0.05 | -0.14 | 0.02 | 0.03 | 0.56 | 0.42 |
| Question_29 | 0.04 | -0.05 | -0.01 | -0.12 | 0.05 | 0.80 | 0.02 |
| Question_49 | 0.01 | 0.00 | 0.81 | 0.07 | 0.06 | -0.25 | 0.33 |
| Question_50 | -0.11 | 0.22 | 0.68 | -0.13 | 0.01 | 0.12 | -0.15 |
| Question_51 | 0.28 | -0.30 | 0.45 | -0.11 | -0.06 | -0.15 | -0.27 |
| Question_52 | 0.03 | 0.05 | 0.65 | -0.05 | 0.02 | 0.08 | -0.28 |
| Question_53 | 0.10 | 0.09 | 0.23 | -0.50 | -0.10 | 0.28 | 0.13 |
| Question_54 | -0.06 | 0.37 | -0.01 | 0.31 | -0.05 | 0.42 | -0.26 |
| Question_55 | 0.02 | 0.79 | -0.21 | -0.01 | 0.03 | 0.06 | 0.15 |
| Question_56 | 0.00 | 0.66 | 0.16 | 0.00 | -0.03 | 0.16 | -0.10 |
| Question_57 | 0.11 | 0.01 | 0.08 | 0.87 | -0.14 | -0.01 | 0.11 |
| Question_58 | -0.09 | 0.67 | 0.26 | 0.09 | 0.15 | -0.04 | -0.34 |
| Question_59 | -0.07 | 0.27 | -0.13 | 0.62 | 0.21 | -0.12 | -0.18 |
| Question_60 | -0.12 | 0.45 | -0.03 | 0.26 | 0.12 | 0.06 | -0.24 |
| Question_61 | -0.05 | 0.69 | -0.01 | 0.11 | -0.03 | 0.03 | 0.04 |
| Question_62 | -0.08 | 0.82 | 0.07 | 0.13 | -0.04 | 0.08 | 0.17 |
| Question_63 | 0.07 | 0.97 | 0.00 | -0.09 | -0.02 | -0.13 | -0.01 |
| Question_64 | 0.03 | 0.19 | 0.21 | 0.04 | 0.01 | 0.09 | 0.34 |
| Question_65 | -0.01 | 0.08 | -0.04 | -0.30 | 0.57 | -0.07 | -0.16 |

Table S6 Summary of loadings and cumulative variances for medicine students’ questions

|  | **PA1** | **PA2** | **PA3** | **PA4** | **PA5** | **PA6** | **PA7** |
| --- | --- | --- | --- | --- | --- | --- | --- |
| SS loadings | 4.26 | 5.06 | 3.17 | 2.55 | 2.72 | 2.48 | 2.52 |
| Proportion Var | 0.11 | 0.13 | 0.08 | 0.07 | 0.07 | 0.06 | 0.06 |
| Cumulative Var | 0.24 | 0.13 | 0.32 | 0.46 | 0.39 | 0.58 | 0.52 |
| Proportion Explained | 0.19 | 0.22 | 0.14 | 0.11 | 0.12 | 0.11 | 0.11 |
| Cumulative Proportion | 0.41 | 0.22 | 0.55 | 0.78 | 0.67 | 1.00 | 0.89 |

Table S7 Correlation coefficients between factors for medicine students’ questions

|  | **PA2** | **PA1** | **PA3** | **PA5** | **PA4** | **PA7** | **PA6** |
| --- | --- | --- | --- | --- | --- | --- | --- |
| **PA2** | 1.00 | -0.03 | 0.11 | 0.06 | 0.16 | -0.17 | 0.16 |
| **PA1** | -0.03 | 1.00 | 0.25 | 0.21 | 0.13 | 0.30 | 0.20 |
| **PA3** | 0.11 | 0.25 | 1.00 | 0.04 | 0.11 | 0.11 | 0.08 |
| **PA5** | 0.06 | 0.21 | 0.04 | 1.00 | 0.11 | 0.15 | 0.21 |
| **PA4** | 0.16 | 0.13 | 0.11 | 0.11 | 1.00 | 0.13 | 0.20 |
| **PA7** | -0.17 | 0.30 | 0.11 | 0.15 | 0.13 | 1.00 | 0.10 |
| **PA6** | 0.16 | 0.20 | 0.08 | 0.21 | 0.20 | 0.10 | 1.00 |

Table S8 Which questions belong to each factor for medicine students’ questions

| **Factors** | **Questions** |
| --- | --- |
| 1 | Question_16, Question_18, Question_19, Question_20, Question_21, Question_24, Question_17 |
| 2 | Question_63, Question_62, Question_55, Question_61, Question_58, Question_56, Question_60, Question_22 |
| 3 | Question_49, Question_50, Question_52, Question_23, Question_51 |
| 4 | Question_57, Question_59, Question_53, Question_26 |
| 5 | Question_14, Question_8, Question_65, Question_11, Question_12, Question_9, Question_13, Question_10 |
| 6 | Question_29, Question_28, Question_54, Question_27 |
| 7 | Question_25, Question_15, Question_64 |

Table S9 Factor loadings for the five-factor model for pharmacy students’ questions

|  | **PA1** | **PA2** | **PA3** | **PA4** | **PA5** |
| --- | --- | --- | --- | --- | --- |
| Question_30 | 0.42 | 0.13 | 0.36 | -0.01 | 0.08 |
| Question_31 | 0.40 | 0.18 | 0.26 | -0.04 | 0.17 |
| Question_32 | 0.19 | 0.21 | 0.14 | 0.02 | 0.68 |
| Question_33 | 0.16 | 0.12 | -0.07 | 0.15 | 0.68 |
| Question_34 | 0.49 | 0.09 | 0.02 | 0.00 | 0.46 |
| Question_35 | 0.42 | 0.10 | 0.16 | -0.14 | 0.55 |
| Question_36 | 0.61 | 0.09 | 0.15 | 0.14 | 0.08 |
| Question_37 | 0.42 | 0.18 | 0.44 | -0.02 | -0.10 |
| Question_38 | 0.69 | 0.07 | 0.04 | 0.10 | 0.08 |
| Question_39 | 0.26 | 0.19 | 0.31 | -0.20 | 0.31 |
| Question_40 | 0.26 | 0.24 | 0.40 | 0.06 | -0.06 |
| Question_41 | 0.27 | -0.09 | 0.64 | -0.34 | 0.13 |
| Question_42 | -0.10 | 0.00 | 0.82 | 0.22 | -0.07 |
| Question_43 | -0.08 | 0.05 | 0.60 | -0.23 | 0.04 |
| Question_44 | 0.83 | 0.03 | -0.06 | 0.11 | 0.01 |
| Question_45 | 0.78 | -0.10 | 0.12 | -0.02 | -0.01 |
| Question_46 | 0.52 | -0.01 | -0.05 | -0.45 | 0.27 |
| Question_47 | 0.71 | -0.01 | -0.04 | -0.24 | 0.18 |
| Question_48 | -0.16 | -0.16 | 0.56 | 0.27 | 0.19 |
| Question_49 | 0.28 | -0.10 | 0.11 | 0.63 | -0.02 |
| Question_50 | 0.15 | 0.07 | 0.12 | 0.40 | 0.15 |
| Question_51 | -0.34 | -0.23 | 0.30 | 0.25 | 0.36 |
| Question_52 | -0.11 | 0.20 | 0.13 | 0.29 | 0.13 |
| Question_53 | 0.25 | 0.32 | 0.01 | 0.24 | -0.53 |
| Question_54 | 0.50 | 0.22 | -0.18 | 0.27 | -0.29 |
| Question_55 | 0.14 | 0.78 | -0.01 | -0.23 | -0.13 |
| Question_56 | -0.05 | 0.68 | -0.08 | -0.34 | -0.03 |
| Question_57 | -0.14 | 0.90 | -0.06 | 0.07 | 0.20 |
| Question_58 | 0.00 | 0.68 | -0.05 | -0.15 | 0.10 |
| Question_59 | 0.19 | 0.76 | -0.01 | -0.10 | -0.10 |
| Question_60 | -0.04 | 0.76 | -0.03 | 0.21 | 0.16 |
| Question_61 | 0.00 | 0.87 | -0.01 | 0.15 | 0.12 |
| Question_62 | 0.04 | 0.70 | 0.15 | 0.33 | -0.05 |
| Question_63 | 0.02 | 0.71 | 0.24 | -0.22 | -0.28 |
| Question_64 | 0.50 | -0.11 | -0.17 | 0.21 | -0.05 |
| Question_65 | 0.22 | -0.07 | -0.01 | 0.21 | -0.42 |

Table S10 Summary of loadings and cumulative variances for pharmacy students’ questions

|  | **PA1** | **PA2** | **PA3** | **PA4** | **PA5** |
| --- | --- | --- | --- | --- | --- |
| SS loadings | 5.58 | 6.12 | 3.04 | 2.06 | 2.91 |
| Proportion Var | 0.15 | 0.17 | 0.08 | 0.06 | 0.08 |
| Cumulative Var | 0.32 | 0.17 | 0.41 | 0.55 | 0.49 |
| Proportion Explained | 0.28 | 0.31 | 0.15 | 0.10 | 0.15 |
| Cumulative Proportion | 0.59 | 0.31 | 0.75 | 1.00 | 0.90 |

Table S11 Correlation coefficients between factors for pharmacy students’ questions

|  | **PA2** | **PA1** | **PA3** | **PA5** | **PA4** |
| --- | --- | --- | --- | --- | --- |
| **PA2** | 1.00 | 0.32 | 0.02 | 0.10 | -0.04 |
| **PA1** | 0.32 | 1.00 | 0.21 | 0.25 | -0.11 |
| **PA3** | 0.02 | 0.21 | 1.00 | 0.14 | -0.02 |
| **PA5** | 0.10 | 0.25 | 0.14 | 1.00 | -0.05 |
| **PA4** | -0.04 | -0.11 | -0.02 | -0.05 | 1.00 |

Table S12 Which questions belong to each factor for pharmacy students’ questions

| **Factors** | **Questions** |
| --- | --- |
| 1 | Question_44, Question_45, Question_47, Question_38, Question_36, Question_46, Question_64, Question_54, Question_34, Question_30, Question_31 |
| 2 | Question_57, Question_61, Question_55, Question_60, Question_59, Question_63, Question_62, Question_56, Question_58 |
| 3 | Question_42, Question_41, Question_43, Question_48, Question_37, Question_40, Question_39 |
| 4 | Question_49, Question_50 |
| 5 | Question_33, Question_32, Question_35, Question_53, Question_65, Question_51 |

Table S13 Cronbach’s alpha test results for medicines and pharmacy students’ questions

| **Medicine students’ questions** | | | **Pharmacy students’ questions** | | |
| --- | --- | --- | --- | --- | --- |
| Factor | No of questions contained in the factor | Consistency test results | Factor | No of questions contained in the factor | Consistency test results |
| 1 | 7 | 0.83 | 1 | 11 | 0.81 |
| 2 | 8 | 0.75 | 2 | 9 | 0.79 |
| 3 | 5 | 0.65 | 3 | 7 | 0.69 |
| 4 | 4 | 0.37 | 4 | 2 | 0.55 |
| 5 | 8 | 0.79 | 5 | 6 | 0.31 |
| 6 | 4 | 0.49 |  | | |
| 7 | 3 | 0.56 |  |  |  |

Figure S1 Overview of responses across faculties for preparedness to assess the need for antibiotics

| Medicine  *% of respondents* | Pharmacy |
| --- | --- |


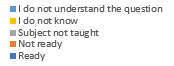


*% of respondents*

Figure S2 Overview of responses across faculties for preparedness to make decisions on initiating antibiotic treatment

| Medicine | Pharmacy |
| --- | --- |


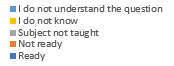


*% of respondents*

*% of respondents*

Figure S3 Overview of responses across faculties for preparedness to monitor antibiotic therapy and assessing emerging evidence

| Medicine | Pharmacy |
| --- | --- |


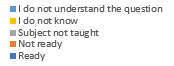


*% of respondents*

*% of respondents*

Figure S4 Overview of responses across faculties for preparedness on antibiotic resistance

*% of respondents*

| Medicine  *% of respondents* | Pharmacy |
| --- | --- |


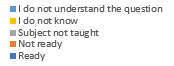


Figure S5 Overview of responses across faculties for preparedness for communication and engagement activities related questions

| Medicine | Pharmacy |
| --- | --- |


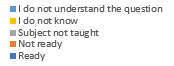


*% of respondents*

*% of respondents*

Figure S6 Correlations between questions relevant for medicine and pharmacy students

|  |  |
| --- | --- |


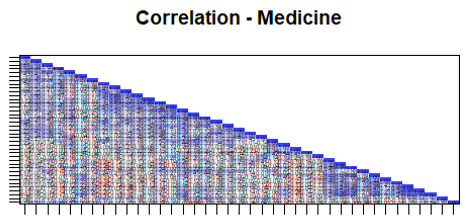

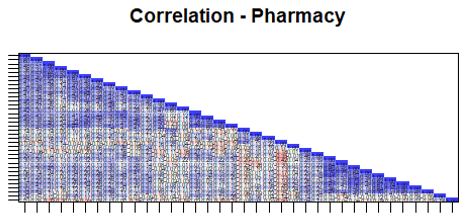


Figure S7 Overview of Eigenvalues of factors for medicine and pharmacy students

| **Medicine** | **Pharmacy** |
| --- | --- |


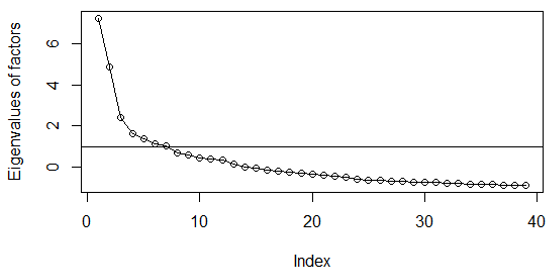

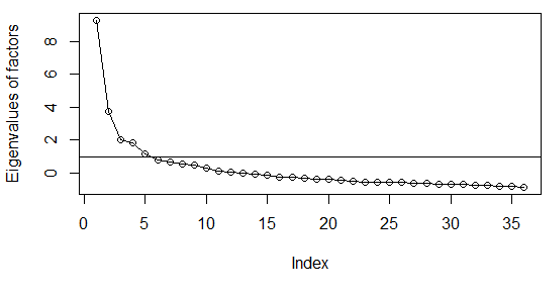


Figure S8 Survey text and questionnaire

**Survey intro message and questionnaire**

Thank you for considering participating in this anonymous survey!

The survey is undertaken to learn more about how future medical and pharmaceutical professionals feel prepared, see their future professional role, and are educated about antibiotic prescribing and counseling in Romania. The survey also seeks to capture their opinions on the willingness to participate in community activities aimed at promoting the appropriate administration of antibiotics and recommendations related to what could be improved in Romania in this field. The results will also be used to validate the proposed questionnaire, so that it can be used in future iterations of this survey and enable standardised data comparison from year to year or between different geographical areas (or countries). Please consider completing this survey if you are either a medicine or a pharmacy student who will graduate and enter the workforce in the coming year.

The data analysis also aims to propose improvements to existing ways of teaching about antibiotics and related public health campaigns (interventions) in Romania. The results will be published in national and international peer-reviewed journals. This study is part of a PhD thesis conducted at Karolinska Institutet, Sweden. If you have any questions about this research, please contact the principal researcher at the email address: ioana.ghiga@ki.se or by phone at 0731 698 587.

The survey is voluntary, completion will be done anonymously and participants will not receive any compensation for their participation. Participants may withdraw from the survey at any time without giving any reason and without any negative consequences. The data collected will be used for research purposes only and will be treated confidentially.

By filling in this survey and submitting your responses, it is understood that you consent to participate in this survey.

It should take you about ten minutes to complete this survey; to be able to use the data in a meaningful way please try to answer all questions – however, you may skip any questions you don’t feel comfortable answering.

**Demographics**

1. How old are you? ________________________

2. Do you have Romanian citizenship?

Yes  No  (please write the country of your citizenship) ___________

3. Do you plan on graduating your studies in either medicine or pharmacy soon (next school year)?

Yes  No

*Please note that this study is aimed for students that will graduate and enter the workforce in the coming year.*

4. Which university are you studying at?

Western University "Vasile Goldiș" Arad

Transilvania University Brașov

"Carol Davila" University of Medicine and Pharmacy Bucharest

"Titu Maiorescu" University Bucharest

University of Medicine and Pharmacy "Iuliu Hațieganu" Cluj-Napoca

"Ovidius" University Constanța

University of Medicine and Pharmacy Craiova

Faculty of Medicine and Pharmacy "Dănărea de Jos" Galati

University of Medicine and Pharmacy "Grigore T. Popa" Iasi

Oradea University

"Lucian Blaga" University Sibiu

University of Medicine, Pharmacy, Sciences and Technology "George Emil Palade" Târgu Mureș

"Victor Babeș" University of Medicine and Pharmacy, Timișoara

5. Which field are you studying in?

Medicine Pharmacy

Based on this answer the respondents will fill in the respective technical areas for *Medicine students* or for *Pharmacy students.*

**Technical preparedness**

In this section we are trying to understand how you feel you are prepared as a future medical professional when it comes to antibiotic use. For each question, please choose one of the answers that match how you feel presently about that technical area. If there are areas that you have not had any teaching, please select ‘subject not taught’.

**I. Medical students – technical areas of preparedness**

6. Assessing the need for antibiotics

| I feel ready to: | Subject not taught | I don't feel ready at all | I don't feel quite ready | I feel ready | I feel very ready | I don’t know | I don’t understand the question |
| --- | --- | --- | --- | --- | --- | --- | --- |
| Understand a clinical situation where the administration of antibiotics may be necessary |  |  |  |  |  |  |  |
| Assess the clinical severity of an infection |  |  |  |  |  |  |  |
| Interpret biomedical markers of inflammation |  |  |  |  |  |  |  |
| Decide on the need for an antibiogram |  |  |  |  |  |  |  |
| Interpret microbiological investigations |  |  |  |  |  |  |  |

7. Decision to initiate antibiotic treatment

| I feel ready to: | Subject not taught | I don't feel ready at all | I don't feel quite ready | I feel ready | I feel very ready | I don’t know | I don’t understand the question |
| --- | --- | --- | --- | --- | --- | --- | --- |
| Differentiate between bacterial colonization and infection |  |  |  |  |  |  |  |
| Differentiate between bacterial and viral upper respiratory tract infections |  |  |  |  |  |  |  |
| Decide on appropriate empiric treatment without using guidelines |  |  |  |  |  |  |  |
| Establish the appropriate timing and duration of antibiotic administration (e.g. based on the urgency of different scenarios or specific infection characteristics) |  |  |  |  |  |  |  |
| Prescribe antibiotics in line with national guidelines |  |  |  |  |  |  |  |
| Assess potential antibiotic allergies or other antibiotic-related adverse events |  |  |  |  |  |  |  |
| Prescribe a combination of antibiotics with other medications |  |  |  |  |  |  |  |

8. Monitoring antibiotic therapy and assessing emerging evidence

| I feel ready to: | Subject not taught | I don't feel ready at all | I don't feel quite ready | I feel ready | I feel very ready | I don’t know | I don’t understand the question |
| --- | --- | --- | --- | --- | --- | --- | --- |
| Decide on continuation or discontinuation of antibiotic treatment in an optimal timeframe based on emerging evidence and developments (e.g. clinical progress and analysis results) |  |  |  |  |  |  |  |
| Investigate the causes of potential failures of antibiotic treatments |  |  |  |  |  |  |  |
| Decide on changing the route of antibiotic administration (for example, from IV to oral) |  |  |  |  |  |  |  |
| Participate as a researcher in clinical or pidemiological (public health) research on antibiotics |  |  |  |  |  |  |  |
| Interpret the findings of scientific studies on antibiotics |  |  |  |  |  |  |  |

9. Antibiotic resistance

| I feel ready to: | Subject not taught | I don't feel ready at all | I don't feel quite ready | I feel ready | I feel very ready | I don’t know | I don’t understand the question |
| --- | --- | --- | --- | --- | --- | --- | --- |
| Use the knowledge gained about the mechanisms of antibiotic resistance formation to try to mitigate this phenomenon |  |  |  |  |  |  |  |
| Engage in discussions with my peers, or other colleagues, about the specific negative consequences of inappropriate antibiotic use, including using recent epidemiological data from Romania |  |  |  |  |  |  |  |

10. Communication and engagement

| I feel ready to: | Subject not taught | I don't feel ready at all | I don't feel quite ready | I feel ready | I feel very ready | I don’t know | I don’t understand the question |
| --- | --- | --- | --- | --- | --- | --- | --- |
| Discuss in lay-man’s terms appropriate antibiotic use with patients, especially in situations when these may not be necessary |  |  |  |  |  |  |  |
| Justify and maintain my position in situations where I may feel coherced (either by patients, senior colleagues or other colleagues) to prescribe antibiotics when I consider these are not needed |  |  |  |  |  |  |  |
| Work in a multidisciplinary team in a clinical setting |  |  |  |  |  |  |  |

**II. Pharmacy students – technical areas of preparedness**

6. Assessing the need for antibiotics

| I feel ready to: | Subject not taught | I don't feel ready at all | I don't feel quite ready | I feel ready | I feel very ready | I don’t know | I don’t understand the question |
| --- | --- | --- | --- | --- | --- | --- | --- |
| Understand a clinical situation where the administration of antibiotics may be necessary |  |  |  |  |  |  |  |
| Based on the physician’s prescription understand why a certain antibiotic was prescribed |  |  |  |  |  |  |  |
| Advise the patient how to manage the symptoms without antibiotics |  |  |  |  |  |  |  |
| Offer recommendations on intermediate drug alternatives, which do not involve antibiotics, and advise the patient if it is necessary to see a doctor immediately |  |  |  |  |  |  |  |

7. Decision to initiate antibiotic treatment

| I feel ready to: | Subject not taught | I don't feel ready at all | I don't feel quite ready | I feel ready | I feel very ready | I don’t know | I don’t understand the question |
| --- | --- | --- | --- | --- | --- | --- | --- |
| Assess the seriousness of a clinical situation and establish whether emergency antibiotic should be given to the patient |  |  |  |  |  |  |  |
| Make recommendations about over-the-counter (OTC) medications that are appropriate for optimizing antibiotic use |  |  |  |  |  |  |  |
| Evaluate potential antibiotic allergies or other antibiotic-related adverse events |  |  |  |  |  |  |  |
| Understand the effects of drug interactions and potential adverse effects of some of these drug combinations |  |  |  |  |  |  |  |
| Offer guidance to patients on infection prevention and spread (for example: advice about the benefits of vaccination, hygiene and hand washing) |  |  |  |  |  |  |  |

8. Monitoring antibiotic therapy and assessing emerging evidence

| I feel ready to: | Subject not taught | I don't feel ready at all | I don't feel quite ready | I feel ready | I feel very ready | I don’t know | I don’t understand the question |
| --- | --- | --- | --- | --- | --- | --- | --- |
| Recommend that the patient continue or stop antibiotic treatment and refer him to a doctor depending on the patient's clinical presentation |  |  |  |  |  |  |  |
| Understand the causes of potential antibiotic treatment failures |  |  |  |  |  |  |  |
| Provide expert advice to healthcare professionals on antibiotic choice, dose, duration and dose adjustment |  |  |  |  |  |  |  |
| Participate as a researcher in clinical or epidemiological (public health) research on antibiotics |  |  |  |  |  |  |  |
| Interpret the findings of scientific studies about antibiotics |  |  |  |  |  |  |  |

9. Antibiotic resistance

| I feel ready to: | Subject not taught | I don't feel ready at all | I don't feel quite ready | I feel ready | I feel very ready | I don’t know | I don’t understand the question |
| --- | --- | --- | --- | --- | --- | --- | --- |
| Use the knowledge gained about the mechanisms of antibiotic resistance formation to try to mitigate this phenomenon |  |  |  |  |  |  |  |
| Engage in discussions with my peers, or other colleagues, about the specific negative consequences of inappropriate antibiotic use, including using recent epidemiological data from Romania |  |  |  |  |  |  |  |

10. Communication and engagement

| I feel ready to: | Subject not taught | I don't feel ready at all | I don't feel quite ready | I feel ready | I feel very ready | I don’t know | I don’t understand the question |
| --- | --- | --- | --- | --- | --- | --- | --- |
| Discuss in lay-man’s terms with patients appropriate antibiotic use, especially in situations when these may not be necessary |  |  |  |  |  |  |  |
| Justify and maintain my position in situations where I may feel coherced (either by patients, senior colleagues or other colleagues) to release antibiotics without prescriptions when I consider them unnecessary |  |  |  |  |  |  |  |
| Work in a multidisciplinary team in a clinical setting |  |  |  |  |  |  |  |

**Engagement willingness**

In this section we are trying to understand how you feel about engaging in different activities related to antibiotic usage.

11. Engagement willingness

| I am willing to: | I'm not willing at all | I'm not really willing | I am willing | I am very willing | I don’t know | I don’t understand the question |
| --- | --- | --- | --- | --- | --- | --- |
| Participate in national awareness campaigns regarding the use of antibiotics by disseminating materials provided by the public health departments |  |  |  |  |  |  |
| Offer community interventions such as workshops for high school students (or younger university students) on appropriate antibiotic use in my spare time |  |  |  |  |  |  |
| Be interested in aspects of antibiotic use as the main topic of my graduate thesis in the faculty I am pursuing |  |  |  |  |  |  |
| Engage in on-my-own study efforts to learn more about antibiotic use |  |  |  |  |  |  |

**Expectations**

In this section we are trying to capture your recommendations in the area of antibiotic use.

12. Please tell us your advice and recommendations for improving antibiotic use.

b. What are your recommendations for the Ministry of Health to create policies or activities aimed at ensuring the responsible use of antibiotics in Romania?

a. What are your recommendations for the university you attend to further your knowledge of antibiotics?

**Teaching methods used for antibiotic education**

13. Which of the following teaching methods would you prefer for learning about appropriate antibiotic use?

| I consider that the following teaching methods would be useful to acquire and deepen my knowledge about the appropriate use of antibiotics | I don't find it useful at all | I don't think it's very useful | I find it useful | I find it very useful | I don’t know | I don’t understand the question |
| --- | --- | --- | --- | --- | --- | --- |
| Lecture/course (with > 30 students) |  |  |  |  |  |  |
| Small lectures/courses (with <30 students) |  |  |  |  |  |  |
| Seminars to discuss clinical cases |  |  |  |  |  |  |
| Group project relevant to the appropriate use of antibiotics |  |  |  |  |  |  |
| E-learning (learning through the virtual means) |  |  |  |  |  |  |
| Discussions using various scenarios (including assuming certain roles) |  |  |  |  |  |  |
| Internships in clinics, hospitals or pharmacies including patient contact |  |  |  |  |  |  |
| Internships in laboratory (where analysis are being done) |  |  |  |  |  |  |
| Internships in pharmaceutical companies producing or marketing (distributing) antibiotics |  |  |  |  |  |  |
| Internships at the Institute or Directorate of Public Health |  |  |  |  |  |  |
| Teaching with examples of situations experienced by older students or recent graduates |  |  |  |  |  |  |

14. On the whole, as a future healthcare professional, do you feel that you have received adequate training to ensure the appropriate use of antibiotics in your professional areas and competencies?

Yes, definetly

Yes, very likely

I would lean more towards no

Definitely - no

I don’t know

I don’t understand the question

15. How do you think the situation with the use of antibiotics in Romania will evolve?

☐ It will evolve for the better

☐ It will remain the same

☐ It will get worse

☐ I don’t know

☐ I don’t understand the question

**Thank you very much for your time and we wish you the best of luck in completing your studies!**

1. In Romania, consultations to the doctors through the state system are covered by the national health insurance scheme. However, there are also private clinics where consultations are paid by patients. [↑](#footnote-ref-2)
